# 25-Hydroxyvitamin D and TSH as Risk Factors or Prognostic Markers in Thyroid Carcinoma

**DOI:** 10.1371/journal.pone.0164550

**Published:** 2016-10-13

**Authors:** Debora Lucia Seguro Danilovic, Bruno Ferraz-de-Souza, Amanda Wictky Fabri, Nathalie Oliveira Santana, Marco Aurelio Kulcsar, Claudio Roberto Cernea, Suemi Marui, Ana Oliveira Hoff

**Affiliations:** 1 Endocrinology, Instituto do Câncer do Estado de São Paulo, Sao Paulo, Brazil; 2 Laboratório de Endocrinologia Celular e Molecular (LIM25), Faculdade de Medicina da Universidade de São Paulo, Sao Paulo, Brazil; 3 Laboratório de Investigação Médica 18 (LIM-18), Faculdade de Medicina da Universidade de São Paulo, Sao Paulo, Brazil; 4 Head and Neck Surgery, Instituto do Câncer do Estado de São Paulo, São Paulo, Brazil; Universite Libre de Bruxelles, BELGIUM

## Abstract

**Objective:**

The increasing incidence of thyroid nodules demands identification of risk factors for malignant disease. Several studies suggested the association of higher TSH levels with cancer, but influence of 25-hydroxyvitamin D (25OHD) is controversial. This study aimed to identify the relationship of thyroid cancer with higher TSH levels and hypovitaminosis D and to evaluate their influence on prognostic characteristics of papillary thyroid carcinomas (PTC).

**Methods:**

We retrospectively evaluated 433 patients submitted to thyroidectomy for thyroid nodules. Patients were categorized according to quartiles of TSH and 25OHD levels. Clinicopathological features were analyzed.

**Results:**

Subjects with thyroid carcinomas were more frequently male and younger compared to those with benign disease. Their median TSH levels were higher and adjusted odds-ratio (OR) for cancer in the highest-quartile of TSH (> 2.4 mUI/mL) was 2.36 (1.36–4.09). Although vitamin D deficiency/insufficiency was prevalent in our cohort (84%), no significant differences in 25OHD levels or quartile distribution were observed between benign and malignant cases. Among 187 patients with PTC, analyses of prognostic features revealed increased risk of lymph nodes metastases for subjects with highest-quartile TSH levels (OR = 3.7, p = 0.029). Decreased 25OHD levels were not overtly associated with poor prognosis in PTC.

**Conclusions:**

In this cross-sectional cohort, higher TSH levels increased the risk of cancer in thyroid nodules and influenced its prognosis, particularly favoring lymph nodes metastases. On the other hand, no association was found between 25OHD levels and thyroid carcinoma risk or prognosis, suggesting that serum 25OHD determination may not contribute to risk assessment workup of thyroid nodules.

## Introduction

The widespread use of cervical imaging for purposes other than investigation of thyroid disease is responsible for the increasing incidence of thyroid nodules. The development of sensitive diagnostic procedures also contributed to the increased prevalence of differentiated thyroid carcinomas, mainly microcarcinomas; but it could not completely explain the increased diagnosis of papillary carcinomas > 4 cm [1].

Some clinical features have been traditionally related to increased risk of cancer in thyroid nodules, such as increasing age, male sex and history of radiation exposure [2]. Observational studies evaluated the relationship of TSH concentrations and risk of thyroid cancer; in a systematic review and meta-analysis of the subject it has been demonstrated a non-linear relationship. A dose response model predicted a doubling of odds of thyroid cancer between a serum TSH level of 0.65 and 4 mU/liter and within the normal range to an elevated serum TSH level, it predicted a doubling of the odds of thyroid cancer between a serum TSH level of 2.2 and 7 mU/liter [3].

Several extraskeletal actions of vitamin D are under investigation. Vitamin D nuclear receptors (VDR) have been identified in tissues not directly involved with bone and mineral metabolism, including the thyroid, and in several cancers, such as breast, prostate, colon, leukemia and thyroid carcinoma [4–8]. Cancer cells express CYP21B1, the enzyme involved in hydroxylation of 25OHD [9, 10]; therefore, in cancer tissues, calcitriol (1,25OHD) action could be enhanced by local synthesis, which, in turn, would depend on circulating 25OHD levels. Several genomic mechanisms of 1,25OHD action through VDR promoting antineoplastic actions were identified [11–13]. *In vitro* studies, demonstrated that calcitriol in cancer cells suppressed anti-apoptotic BCL2 [14], inhibited prostaglandin synthesis and signaling [15] and, suppressed the expression of VEGF through repression of hypoxia-inducible factor 1 alpha [16]. 1,25OHD also regulates microRNAs involved in carcinogenesis and specific pathways driving colon [17], breast [18] and prostate cancer [19]. Furthermore, vitamin D has been shown to have positive effects on cancer prevention and tumor control in animal models [20, 21]. Some observational and clinical studies suggested an association of vitamin D deficiency and cancer, especially breast, prostate, and colon cancer, and leukemia [22–26]. A few studies have analyzed the association of 25OHD deficiency and thyroid cancer with conflicting results [27–30].

The aims of this study were to identify the association of vitamin D deficiency or higher TSH levels with thyroid cancer and evaluate their influence on prognostic features of papillary thyroid carcinomas.

## Materials and Methods

### Patients

We retrospectively evaluated patients submitted to thyroidectomy due to suspicious nodules or symptomatic goiter in *Instituto do Cancer do Estado de São Paulo (ICESP)* from 2009 to 2012. This study was approved by the Institutional Review Board, *Comitê de Ética em Pesquisa da Faculdade de Medicina da Universidade de São Paulo* (approval number 159/13). Data were analyzed anonymously and retrospectively.

In patients with suspicious nodules by cytology, total thyroidectomy and therapeutic neck dissection was performed if metastasis was suspected by preoperative ultrasound or intraoperative inspection.

Clinical characteristics, demographic data, laboratory results and histological features were retrospectively collected. We selected patients who had serum TSH and 25OHD levels performed within 6 months prior to surgery. Subjects who were on levothyroxine or antithyroid drugs or had received vitamin D supplements were excluded.

Patients with papillary thyroid carcinomas (PTC) were classified into <45 years old and ≥ 45 years old, as thyroid cancers are staged differently depending on age. Histopathological features associated with poor prognosis were registered: increased size, aggressive variant, which included all but classic and follicular ones, multifocality, presence of extrathyroidal extension or vascular invasion and diagnosis of lymph nodes metastasis in initial surgery. [2]. Tumor size was defined as the greatest cancer diameter based on histopathology report. We applied the TNM classification of the American Joint Committee on Cancer [31].

### Methods

Serum TSH levels were measured using commercial fluorimmunoassay (AutoDELFIA^®^, Upsala, Turku, Finland), with normal values from 0.4 to 4.5 mUI/mL. Patients were categorized into quartiles of preoperative TSH levels: quartiles 1, TSH≤0.94 mUI/mL, quartile 2, 0.95–1.58 mUI/mL, quartile 3, 1.59–2.40 mUI/mL, and quartile 4, TSH>2.4 mUI/mL.

Serum 25OHD levels were measured using commercial chemiluminescent immunoassay (DiaSorin Liaison 25 OH Vitamin D Total, Stillwater, MN, USA). Vitamin D deficiency was defined by 25OHD ≤20 ng/mL and insufficiency by 25OHD levels from 21 to 29 ng/mL based on The Endocrine Society Clinical Practice Guideline [32]. Adjustment for seasonal variation in 25OHD levels was based on sampling date: summer, from December to February, autumn, from March to May, winter, from June to August, and spring, from September to November. Patients were categorized into quartiles by preoperative 25OHD levels: quartile 1, 25OHD≤16 ng/mL, quartile 2, 25OHD 17–21 ng/mL, quartile 3, 25OHD 22–27 ng/mL, and quartile 4, 25OHD>27 ng/mL.

### Statistical Analysis

Data were processed using PASW Statistics software version 17.0 (SPSS Inc., Chicago, IL, USA). Two-tailed p-values were used and p-values <0.05 were considered statistically significant.

Categorical variables are presented as absolute and relative (percentages) frequencies. Differences were evaluated by Pearson’s χ^2^-test and Fisher’s exact test when appropriate. Continuous variables are presented as mean ± standard deviation and median values are presented if non-normal distribution. Differences among studied subgroups were determined using Student’s *t*-test if presenting normal distribution, and Mann-Whitney U test for non-normal distributions.

Pearson correlation coefficients were determined among age, TSH levels and 25OHD levels in all subjects and tumor size, TSH levels and 25OHD levels in PTC by linear regression analysis, after logarithmic transformation of non-normal variables.

Logistic regression analysis were used to evaluate the effect of TSH and 25OHD on the risk of cancer and on the aggressiveness of papillary thyroid cancer. Unadjusted odds ratio (OR) and 95% confidence intervals (CI) were calculated with a univariate logistic regression model using the first quartile of TSH and the forth quartile of 25OHD, respectively, as references. Adjusted OR of each TSH quartiles were also calculated after, controlling for sex, age at surgery and body mass index (BMI) in multivariate analysis. Adjusted OR of each 25OHD quartiles were calculated, after controlling for sex, age at surgery, body mass index (BMI), season of the year and TSH quartiles in multivariate analysis.

## Results

From a total of 832 patients submitted to thyroidectomy due to suspicious nodules or symptomatic goiter in our institution from 2009 to 2012, 433 patients met our inclusion criteria and were subsequently analyzed (cohort). There were no differences in gender, age or final diagnosis (benign or malignant thyroid disease) between included and excluded subjects.

Cytological findings prior to surgery were indeterminate or malignant in 81% and 17% of the cohort, respectively. Two hundred and thirty four subjects (54%) had a final diagnosis of benign nodular thyroid disease, including goiter (87% of benign disease), follicular adenomas (10%) and thyroiditis (3%). One hundred and ninety nine patients (46%) had thyroid carcinoma, being 94% papillary and 5% follicular thyroid cancer. Clinical features of patients with benign or malignant thyroid disease are described in Table 1. In the entire cohort, median TSH levels were 1.58 mUI/mL and median 25OHD levels were 21 ng/mL. Vitamin D deficiency was diagnosed in 48% of the cohort and insufficiency was present in 36%. As expected, variations of 25OHD levels were influenced by season, being significantly lower in spring and winter (18.9±6.4 ng/mL and 20.0±8.6 ng/mL, respectively) compared to autumn and summer (24.3±8.7 ng/mL and 21.9±7.6 ng/mL, respectively, p<0.001). Serum 25OHD levels were lower in female patients (21.2±7.9 ng/mL *vs*. 25.7±10.1 ng/mL in male, p = 0.007). In the linear regression analysis, log-transformed 25OHD levels inversely correlated to age (r = -0.135, p = 0.002), but they did not correlate significantly to log-transformed BMI or TSH levels after adjusting for sex, age and season of the year.

**Table 1:** Clinical characteristics of patients with benign thyroid nodules and thyroid carcinomas.

	Benign (n = 234)	Malignant (n = 199)	p
**female/male (%)**	217 / 17 (93 / 7)	171 / 28 (86/14)	0.021
**age years**	54.6±14.5	51.6±15.0	0.038
**BMI kg/m**^**2**^ **(median)**	28.1±5.7 (27.3)	28.2±6.0 (27.5)	0.92
**TSH mUI/mL (median)**	2.1±4.7 (1.4)	2.1±1.6 (1.7)	0.004
**TSH**			
**• Quartile 1 (%)**	64 (27.8)	39 (19.8)	
**• Quartile 2 (%)**	63 (27.3)	47 (23.9)	
**• Quartile 3 (%)**	59 (25.5)	48 (24.4)	
**• Quartile 4 (%)**	45 (19.5)	63 (32)	
**25 hydroxyvitamin D ng/mL (median)**	22.0±8.5 (21.3)	21.3±8.0 (20.0)	0.30
**25OHD**			
**• Quartile 1 (%)**	50 (21.4)	55 (27.6)	
**• Quartile 2 (%)**	55 (23.5)	48 (24.1)	
**• Quartile 3 (%)**	71 (30.3)	42 (21.1)	
**• Quartile 4 (%)**	58 (24.8)	54 (27.1)	

### Benign Thyroid Nodular Diseases vs. Thyroid Carcinomas

Patients with thyroid carcinomas were more frequently male (14% *vs*. 7%, p = 0.021) and younger (mean age 51.6±15.0 *vs*. 54.6±14.5, p = 0.038) than patients with benign thyroid disease (Table 1).

**Fig 1:**
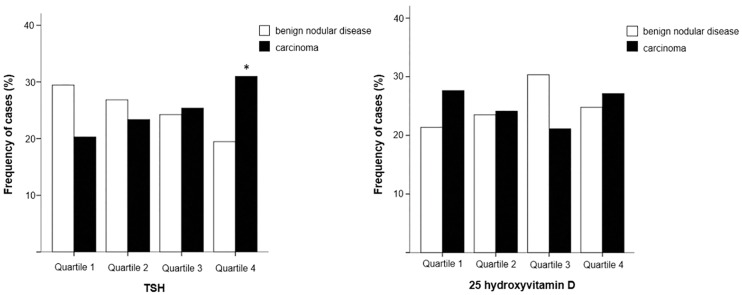
Serum TSH and 25-hydroxyvitamin D levels in patients with thyroid nodules. (a) Serum TSH level distribution in benign nodular thyroid disease and thyroid carcinomas. (b) Serum 25-hydroxyvitamin D level distribution in benign nodular thyroid disease and thyroid carcinomas. Benign nodular thyroid diseases (*white boxes*), thyroid carcinomas (*black boxes*), * p = 0.02

Median TSH levels were higher in patients with carcinoma compared to benign disease (Table 1). Highest-quartile TSH levels were significantly more frequent in malignant cases compared to benign ones (32% *vs*. 19.5%, p = 0.02) (Fig 1a). Unadjusted odds-ratio (OR) for carcinoma in the subgroup with TSH levels in the 4^th^ quartile in relation to subgroups with lower TSH levels was 2.3 (CI 1.33–3.99, p = 0.003). After adjusting for sex, age and BMI, TSH > 2.4 mUI/mL (4^th^ quartile) remained a risk factor for malignant disease (OR 2.36, CI 1.36–4.09, p = 0.002).

No significant differences in serum 25OHD levels were observed between subjects with benign and malignant thyroid disease. Vitamin D deficiency was present in 44.9% of the benign nodules and 51.8% of the thyroid carcinomas (p = 0.153), and quartile distribution of 25OHD levels did not differ significantly between groups (Fig 1b), even after adjusting for sex, age, BMI, season of the year and TSH quartile. Adjusted OR for cancer in the subgroup with 25OHD levels in the 1^st^ quartile in relation to subgroups with higher 25OHD levels was 1.18 (CI95% 0.70–2.01, p = 0.54).

### Papillary Thyroid Carcinomas

Clinical and pathological characteristics associated with poor prognosis in 187 patients with papillary thyroid carcinomas (PTC) were analyzed (Table 2). Median TSH levels were higher in PTCs with lymph nodes metastasis (N1 2.3 mUI/mL *vs*. N0 1.7 mUI/mL, p = 0.018). Unadjusted OR for lymph nodes metastases in the subgroup with TSH levels in the 4^th^ quartile in relation to subgroups with lower TSH levels was 3.71 (CI 1.14–12.05, p = 0.029), and it remained the same after adjusting for sex, age and BMI.

**Table 2:** Adjusted Odds Ratios and confidence intervals of TSH quartiles and 25 hydroxyvitamin D quartiles for various prognostic factors in papillary thyroid carcinomas.

	TSH							25 hydroxyvitamin D						
	Quartile 1 (n = 36)	Quartile 2 (n = 45)		Quartile 3 (n = 44)		Quartile 4 (n = 60)		Quartile 1 (n = 50)		Quartile 2 (n = 47)		Quartile 3 (n = 42)		Quartile 4 (n = 48)
	OR (CI)	OR (CI)	p	OR (CI)	p	OR (CI)	p	OR (CI)	p	OR (CI)	p	OR (CI)	p	OR (CI)
**Male**	1*	1.45 (0.32–6.53)	0.63	2.03 (0.49–8.50)	0.33	2.08 (0.53–8.26)	0.30	0.38 (0.11–1.32)	0.13	0.50 (0.16–1.63)	0.25	0.70 (0.23–2.18)	0.54	1*
**≥ 45 years**	1*	1.05 (0.42–2.63)	0.92	1.12 (0.45–2.79)	0.81	1.20 (0.50–2.89)	0.68	2.89 (1.17–7.13)	0.021	2.42 (1.00–5.90)	0.051	0.82 (0.35–1.88)	0.63	1*
**Tumor > 1cm**	1*	1.47 (0.61–3.55)	0.40	1.34 (0.56–3.23)	0.51	2.23 (0.96–5.18)	0.061	0.39 (0.17–0.89)	0.025	0.35 (0.15–0.81)	0.014	0.63 (0.27–1.48)	0.28	1*
**Aggressive variant**	1*	0.54 (0.09–3.42)	0.51	1.08 (0.23–5.16)	0.92	0.41 (0.06–2.55)	0.34	0.96 (0.06–15.8)	0.96	4.28 (0.46–39.8)	0.20	6.22 (0.70–55.6)	0.10	1*
**Multifocality**	1*	1.34 (0.55–3.24)	0.52	1.47 (0.61–3.52)	0.39	1.04 (0.45–2.40)	0.94	0.92 (0.41–2.06)	0.85	0.84 (0.38–1.90)	0.68	0.64 (0.28–1.50)	0.31	1*
**ETE**	1*	1.16 (0.47–2.88)	0.75	1.19 (0.48–2.91)	0.71	1.61 (0.69–3.76)	0.27	1.11 (0.49–2.52)	0.80	0.91 (0.40–2.11)	0.83	1.47 (0.63–3.41)	0.38	1*
**VI**	1*	2.14 (0.51–8.96)	0.30	1.38 (0.31–6.20)	0.67	1.81 (0.45–7.33)	0.40	1.64 (0.50–5.43)	0.42	0.18 (0.02–1.63)	0.13	2.29 (0.70–7.49)	0.17	1*
**LN**	1*	2.43 (0.69–8.51)	0.17	1.48 (0.40–5.51)	0.56	3.71 (1.14–12.1)	0.029	0.84 (0.32–2.21)	0.72	0.67 (0.24–1.86)	0.44	1.02 (0.38–2.73)	0.96	1*
**T stages 3/ 4**	1*	1.03 (0.42–2.54)	0.94	1.16 (0.48–2.81)	0.75	1.34 (0.58–3.10)	0.50	1.11 (0.49–2.49)	0.81	0.76 (0.33–1.76)	0.52	1.34 (0.58–3.10)	0.50	1*
**Stages III/IV**	1*	1.31 (0.48–3.52)	0.60	1.23 (0.46–3.29)	0.69	1.29 (0.50–3.30)	0.60	1.74 (0.71–4.26)	0.23	1.25 (0.49–3.17)	0.64	1.16 (0.44–3.05)	0.76	1*

* reference

Lower 25OHD levels were associated with increased age (≥45 years) and tumors > 1 cm (Table 2). Unadjusted OR for tumors > 1cm in the subgroups with 25OHD levels in the 1^st^ and 2^nd^ quartiles in relation to subgroups with higher 25OHD levels were 0.36 (0.16–0.82, p = 0.015) and 0.34 (0.15–0.78, p = 0.011), respectively. After adjusting for sex, age, season of the year, BMI and quartiles of TSH, adjusted ORs were 0.39 (0.17–0.89, p = 0.025) and 0.35 (0.15–0.81, p = 0.014), respectively

However, in linear regression models, log-transformed size did not correlate with log-transformed 25OHD (r = 0.12, p = 0.051) or TSH (r = 0.027, p = 0.36) levels, even after adjusting for age, sex, log-transformed BMI and season of the year.

## Discussion

The increasing diagnosis of thyroid nodules demands identification of risk factors that could guide the need of further diagnostic evaluation in suspicious cases or support clinical follow-up of less suspicious ones.

Several studies associated higher TSH levels with increased risk of thyroid cancer. In their meta-analysis, McLeod *et al*. calculated a pooled OR for higher serum TSH of 1.23 (1.11–1.37) per milliunits per liter, but the authors highlighted the non-linear relationship of TSH and cancer [3]. In our study, median TSH levels were higher in malignant cases; serum levels of TSH > 2.4 mUI/mL (4^th^ quartile) were more frequent in patients with thyroid carcinoma (32%) compared to patients with benign thyroid disease (19.5%, p = 0.02). Risk of thyroid cancer increased 2.4 times for TSH levels in the highest range.

Despite this association, a direct role for TSH in thyroid carcinogenesis and cancer biology remains unclear. TSH acts as a growth factor to thyroid nodules. However, our data does not support a putatively stimulatory effect of TSH in thyroid carcinoma, since TSH levels did not correlate with tumor size in our patients. It is possible that such association is a result of increased cancer detection as higher TSH would favor prior occult microcarcinomas to grow into detectable size [33]. As regards the association of prognostic markers with serum TSH levels, meta-analyses have revealed contradictory data so far [3]. *In vitro* studies suggested TSH stimulation of invasion and growth of follicular cancer cells, particularly by protein kinase C stimulation [33, 34]. Some reports observed increased risk of extrathyroidal extension [33, 35] and lymph nodes metastasis [36, 37] with higher TSH levels. We identified an association of increased TSH levels only with lymph nodes metastasis in PTC. Preoperative TSH levels > 2.4 mUI/mL (4^th^ quartile) increased 3.7 times the risk of lymph nodes metastases (p = 0.029). Jonklaas *et al*. showed a positive correlation of TSH levels and number of metastatic lymph nodes [30].

The role of vitamin D deficiency in carcinogenesis is more controversial, but worthy of investigation considering the alarming worldwide rates of hypovitaminosis D [38]. We observed that vitamin D insufficiency and deficiency were very prevalent in our population (48% and 36%, respectively), especially during winter and spring, showing the striking influence of sunlight exposure to vitamin D status even in a mainly tropical country such as Brazil. Indeed, a recent study in healthy volunteers in the same region reported 77.4% of hypovitaminosis D [39]. Furthermore, we confirmed an inverse correlation of age and 25OHD levels in our population, corresponding to the expected reduced ability of vitamin D skin synthesis in older individuals [38]. We have also observed more prevalent vitamin D deficiency in females, as previously reported [40, 41]. However, 25OHD levels did not correlate significantly with serum TSH levels or, more surprisingly, body mass index (BMI); these findings are in contrast to previous reports [41–44] and warrant further exploration of the Brazilian population in a larger scale.

The association of vitamin D deficiency with cancer is suggested by epidemiologic studies. Meta-analyses have demonstrated a reduction in colon cancer risk in subjects with higher 25OHD compared to lower 25OHD levels [45]. Data from prostate and breast cancer studies are contradictory [46]. A systematic review of prospective studies in prostate cancer failed to demonstrate an inverse correlation with 25OHD levels [46], and one study even suggested an increased risk with higher 25OHD level (> 36 ng/mL). In breast cancer, while a robust case-control study did not show a significant association between cancer risk and 25OHD levels [47], meta-analyzes of prospective studies found a lower risk of breast cancer in post-menopausal women with 25OHD levels between 27 and 35 ng/mL [48].

Data from thyroid cancer studies are also discordant [49–51]. Initial studies suggested seasonal variations in the incidence of thyroid cancer, which could potentially reflect variation in 25OHD levels [52]. A few studies evaluated 25OHD levels and risk of thyroid cancer. Defining vitamin D deficiency by 25OHD levels less than 15 and 20 ng/mL, respectively, Roskies *et al*. [27] and Sahin *et al*. [53] found significant association of hypovitaminosis D with thyroid cancers. Although a large population was analyzed in the latter study (344 PTC *vs*. 112 normal controls), it lacked adjustment for vitamin D supplement intake, seasonal variations, and TSH [53]. Other reports, including a small prospective study, failed to demonstrate an influence of 25OHD levels on thyroid cancer risk [28, 29, 54].

Definitions of vitamin D sufficiency status based on serum 25OHD levels have only been proposed in the context of bone and mineral metabolism [32, 55] Since a 25OHD cut-off level is not well defined for putative extraskeletal actions of vitamin D, we analyzed our patients according to quartiles of 25OHD levels. Seasonal, sex, age and BMI adjustments were necessary to avoid interference in the analyses. Taking all these necessary precautions, we could not demonstrate an association of lower 25-hydroxyvitamin D levels with risk of thyroid carcinoma.

Regarding prognosis, several studies have suggested an inverse association of 25OHD levels with cancer mortality [56], and a low 25OHD level (< 30 ng/mL) has been associated with poor prognostic features in melanomas [57], leukemia [58] and breast cancer [59]. In papillary thyroid carcinomas, Kim *et al*. [60] have observed a significant association between lower 25OHD levels and lymph nodes metastasis in female patients. However, these findings were not reproduced by other groups [54, 61]. We did not observe an association of reduced 25OHD levels and poor prognostic histological features in PTC. In addition, we have also not observed a previously reported correlation of tumor size and 25OHD levels [62]. Indeed, our findings suggest the contrary, since the risk of tumors larger or equal to 1 cm was reduced in individuals with 25OHD levels in the lowest quartiles (OR = 0.39 for 1^st^ and OR = 0.35 for 2^nd^ quartiles).

Despite the large analyzed population, our study has limitations. As a retrospective cross-sectional study, we could not evaluate the impact of vitamin D deficiency treatment on cancer-related outcomes. In addition, our analysis was based on a single TSH and 25OHD measurement, which in some cases happened up to 6 months before surgery. On the other hand, our large cohort has allowed for findings that are more conclusive even after careful exclusion of patients who received any medication that would interfere with this analysis.

In conclusion, in this cross-sectional cohort, higher TSH levels were associated with increased risk of cancer and with increased risk of lymph nodes metastases in papillary thyroid carcinomas. On the other hand, 25OHD levels were not associated with risk or prognosis of thyroid carcinomas, suggesting that monitoring of vitamin D status may not be warranted in the diagnostic routine of nodular thyroid disease.

## Supporting Information

S1 File Patients’ clinical and laboratory data. (XLS)
